# Supplementary material for: A Non-Canonical Teleost NK-Lysin: Antimicrobial Activity via Multiple Mechanisms
Source: Int J Mol Sci. 2022 Oct 22;23(21):12722. doi: 10.3390/ijms232112722 (PMC9654944; doi:10.3390/ijms232112722)
Supplement: Supplementary file 1 [file ijms-23-12722-s001.zip › ijms-1965590-supplementary.pdf]

Supplementary material

Table S1. NKL sequences used for phylogenetic analysis and sequence alignment in this study.

Accession number	Species	Protein
XM_020919797	*Boleophthalmus pectinirostris*	NKL
AGM21637	*Cynoglossus semilaevis*	NKL
XM_026230159	*Carassius auratus*	NKL
KT877168	*Ctenopharyngodon idella*	NKL
NM_001303323	*Larimichthys crocea*	NKL
MF113051	*Lateolabrax japonicus*	NKL
XM_018663395	*Latescal carifer*	NKL
XM_003962706	*Takifugu rubripes*	NKL
KU705506	*Scophthalmus maximus*	NKL
XM_015969930	*Nothobranchius furzeri*	NKL
MF678822	*Oreochromis niloticus*	NKL
XM_020487202	*Oncorhynchus kisutch*	NKL
XM_014129907	*Salmo salar*	NKL
XM_021574735	*Oncorhynchus mykiss*	NKL
XM_004082250	*Oryzias latipes*	NKL
XM_021473084	*Danio rerio*	NKL
KP100115	*Danio rerio*	NKLa
KP100116	*Danio rerio*	NKLb
KP100117	*Danio rerio*	NKLc
KP100118	*Danio rerio*	NKLd
NM_001200208	*Ictalurus punctatus*	NKL1
DQ153186	*Ictalurus punctatus*	NKL2
DQ153187	*Ictalurus punctatus*	NKL3
AU260449	*Paralichthys olivaceus*	NKL
XP_035024225.2	*Hippoglossus stenolepis*	NKL
XP_019940439.1	*Paralichthys olivaceus*	PREDICTED: prosaposin-like
KAF5165287.1	*Hippoglossus stenolepis*	Hypothetical protein
XP_041669156.1	*Cheilinus undulatus*	Antimicrobial peptide NK-lysin
XP_018518922.1	*Lates calcarifer*	PREDICTED: saposin-C-like
XP_035529581.1	*Morone saxatilis*	NK-lysin-like
ALH22547.1	*Larimichthys crocea*	NK-lysin-like type 2 protein
XP_005477177.1	*Oreochromis niloticus*	Antimicrobial peptide NK-lysin

Table S2. Primers used in this study.

Primer	Sequence(5′→3′)
RT-β-Actin-F	AACCGCTGCCTCCTCCTCAT
RT-β-Actin-R	TCGGGACAACGGAACCTCTC
RT-NKLnc-F	TGCGGAATGGAGCATTTCCT
RT-NKLnc-R	TCCATTGCAGGCCTTGTTCA
RT-18S rRNA-F	GGTCTGTGATGCCCTTAGATGTC
RT-18S rRNA-R	AGTGGGGTTCAGCGGGTTAC
RT-α-tubulin-F	TGACATCACAAACGCCTGCTTC
RT-α-tubulin-R	GCACCACATCTCCACGGTACAG
RT-GAPDH-F	CAACGGCGACACTCACTCCTC
RT-GAPDH-R	TCGCAGACACGGTTGCTGTAG
NKLnc-F	ATGAGTCCTGTTTTCAGCATCG
NKLnc-R	CTTTTTGCAGAGTTTCGCTTT

Table S3. The sequences of the siRNAs used for gene knockdown.

SiRNA	Forward primer (5′→3′)	Reverse primer (5′→3′)	Target gene
siR1	GGAACAAACAGAAGAGGAUTT	AUCCUCUUCUGUUUGUUCCTT	NKLnc
siR2	CCCACCAUGAGAAUCCCAATT	UUGGGAUUCUCAUGGUGGGTT	NKLnc
NCS	UUCUCCGAACGUGUCACGUTT	ACGUGACACGUUCGGAGAATT	None

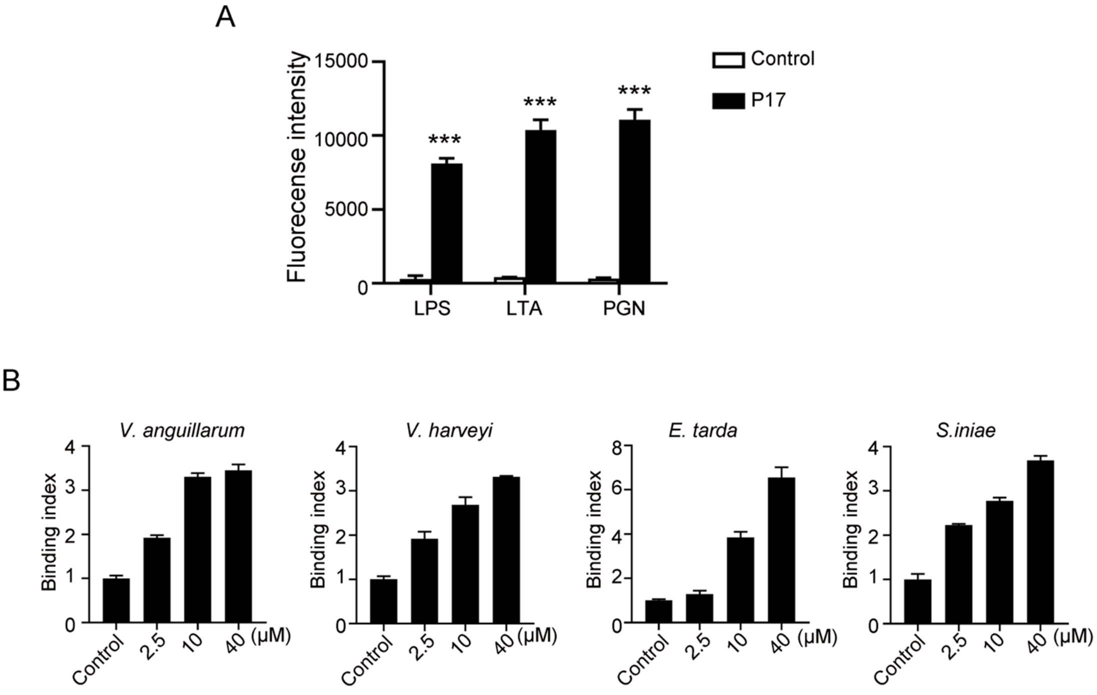

Figure S1. Bacteria binding activity of P17. (A) The binding of P17 to different bacterial cell wall components was determined by ELISA. Values are the means of triplicate expreiments and shown as means ± SD. ***$P < 0.001$. **(B)** Binding of P17 in different doses to bacteria *Vibrio anguillarum, Vibrio harveyi, Edwardsiella tarda* and *Streptococcus iniae* was determined by ELISA.

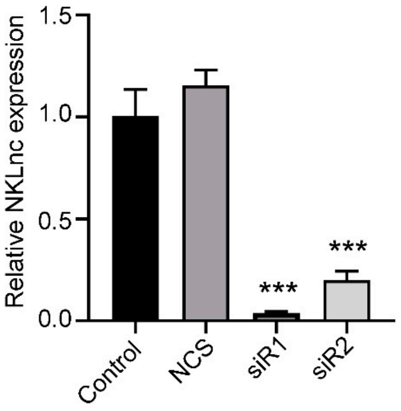

Figure S2. Verification of NKLnc knockdown. (A) FG-9307 cells were treated with or without (control) siR1, siR2, or the negative control siRNA (NCS) for 24 h, and NKLnc expression was determined by RT-qPCR. Values are the means of triplicate expreiments and shown as means ± SD. ****P* $<$ 0.001.
